# Voice-Activated Cognitive Behavioral Therapy for Insomnia: A Randomized Clinical Trial

**DOI:** 10.1001/jamanetworkopen.2024.35011

**Published:** 2024-09-24

**Authors:** Claire M. Starling, Daniel Greenberg, Daniel Lewin, Callen Shaw, Eric S. Zhou, Daniel Lieberman, Jiling Chou, Hannah Arem

**Affiliations:** 1 Implementation Science, Healthcare Delivery Research, MedStar Health Research Institute, Washington, DC; 2 Media Rez, Washington, DC; 3 Sleep Health and Wellness Center, Santa Barbara, California; 4 Department of Psychosocial Oncology and Palliative Care, Dana-Farber Cancer Institute, Boston, Massachusetts; 5 George Washington University, Washington, DC; 6 Center for Biostatistics, Informatics, and Data Science, MedStar Health Research Institute, Hyattsville, Maryland; 7 Department of Oncology, Georgetown University, Washington DC

## Abstract

**Importance:**

Insomnia symptoms affect an estimated 30% to 50% of the 4 million US breast cancer survivors. Previous studies have shown the effectiveness of cognitive behavioral therapy for insomnia (CBT-I), but high insomnia prevalence suggests continued opportunities for delivery via new modalities.

**Objective:**

To determine the efficacy of a CBT-I–informed, voice-activated, internet-delivered program for improving insomnia symptoms among breast cancer survivors.

**Design, Setting, and Participants:**

In this randomized clinical trial, breast cancer survivors with insomnia (Insomnia Severity Index [ISI] score >7) were recruited from advocacy and survivorship groups and an oncology clinic. Eligible patients were females aged 18 years or older who had completed curative treatment more than 3 months before enrollment and had not undergone other behavioral sleep treatments in the prior year. Individuals were assessed for eligibility and randomized between March 2022 and October 2023, with data collection completed by December 2023.

**Intervention:**

Participants were randomized 1:1 to a smart speaker with a voice-interactive CBT-I program or educational control for 6 weeks.

**Main Outcomes and Measures:**

Linear mixed models and Cohen *d* estimates were used to evaluate the primary outcome of changes in ISI scores and secondary outcomes of sleep quality, wake after sleep onset, sleep onset latency, total sleep time, and sleep efficiency.

**Results:**

Of 76 women enrolled (38 each in the intervention and control groups), 70 (92.1%) completed the study. Mean (SD) age was 61.2 (9.3) years; 49 (64.5%) were married or partnered, and participants were a mean (SD) of 9.6 (6.8) years from diagnosis. From baseline to follow-up, ISI scores changed by a mean (SD) of −8.4 (4.7) points in the intervention group compared with −2.6 (3.5) in the control group (*P* < .001) (Cohen *d*, 1.41; 95% CI, 0.87-1.94). Sleep diary data showed statistically significant improvements in the intervention group compared with the control group for sleep quality (0.56; 95% CI, 0.39-0.74), wake after sleep onset (9.54 minutes; 95% CI, 1.93-17.10 minutes), sleep onset latency (8.32 minutes; 95% CI, 1.91-14.70 minutes), and sleep efficiency (−0.04%; 95% CI, −0.07% to −0.01%) but not for total sleep time (0.01 hours; 95% CI, −0.27 to 0.29 hours).

**Conclusions and Relevance:**

This randomized clinical trial of an in-home, voice-activated CBT-I program among breast cancer survivors found that the intervention improved insomnia symptoms. Future studies may explore how this program can be taken to scale and integrated into ambulatory care.

**Trial Registration:**

ClinicalTrials.gov Identifier: NCT05233800

## Key Points

**Question:**

Does a daily, in-home, voice-interactive program delivering cognitive behavioral therapy for insomnia (CBT-I) impact insomnia symptoms among breast cancer survivors?

**Findings:**

In this randomized clinical trial of 76 women, participants who received the CBT-I intervention demonstrated a clinically marked reduction in Insomnia Severity Index scores compared with the control group.

**Meaning:**

The findings suggest that this in-home, voice-interactive CBT-I program has potential for widespread dissemination to address insomnia symptoms.

## Teaser

This randomized clinical trial investigates whether in-home delivery of a daily voice-activated cognitive behavioral therapy program improves insomnia symptoms among survivors of breast cancer.

## Introduction

There were over 4 million breast cancer survivors in the US in 2023.^1^ The cancer-related physiological processes, effects of oncotherapies, induced menopause, comorbid mood disorders, and psychosocial and economic stressors associated with breast cancer treatment are all potential triggers for development or exacerbation of insomnia.^2,3^ Insomnia describes trouble falling asleep, staying asleep, and/or poor-quality sleep that impacts daytime function.^4^ Insomnia is a persistent issue experienced by around 30% to 50% of breast cancer survivors.^5^ Chronic insomnia has been associated with decrements in cardiometabolic and immune system health, neurobehavioral function, depression, fatigue, quality of life, and mortality.^6,7^

Cognitive behavioral therapy for insomnia (CBT-I) is the primary recommended insomnia treatment,^8^ endorsed by the National Comprehensive Cancer Network, American College of Physicians, and American Academy of Sleep Medicine.^8,9,10^ However, in-person CBT-I is not readily accessible due to scarcity of trained practitioners and a 4- to 8-week program that poses engagement challenges.^11^

Digital CBT-I interventions delivered via the internet have demonstrated effectiveness,^12,13,14,15^ addressing some concerns about access. Recent research has also explored the use of voice-activated programs for managing chronic conditions and supporting patients with cancer during chemotherapy,^16,17,18,19,20^ offering new avenues to engage individuals in the home. However, to our knowledge, this technology has not previously been used to deliver CBT-I. Smart speakers might increase accessibility of CBT-I and emulate therapist interactions via voice interactions. Feasibility trial results suggested that breast cancer survivors with insomnia favorably responded to a smart speaker program.^21^ Therefore, our objective was to test the impact of a 6-week, in-home, daily CBT-I smart speaker program (intervention) compared with web-based educational content (control). We hypothesized we would see greater improvement in insomnia symptoms on the Insomnia Severity Index (ISI) and on sleep diaries in the intervention group compared with the control group.

## Methods

Study methods and reporting of this randomized clinical trial followed the Consolidated Standards of Reporting Trials (CONSORT) reporting guideline.^21^ This study was approved by the MedStar-Georgetown institutional review board and was registered on ClinicalTrials.gov (NCT05233800). There were no changes to the protocol (Supplement 1) after registration. Informed consent was obtained from study participants verbally and documented in the MedStar Health Research Institute REDCap platform by the person obtaining consent.

Most participants were recruited through the Love Research Army of the Dr Susan Love Foundation and local survivorship groups. We also recruited from an urban breast medical oncology clinic with a large proportion of Black or African American participants, focusing on women attending survivorship visits. Eligible participants were aged 18 years or older, self-reported or had a documented diagnosis of breast cancer, were female, completed curative treatment (surgery, radiation, chemotherapy, and/or immunotherapy) more than 3 months prior to enrollment, agreed to consistent use of prescribed sleep and/or psychiatric medications during the study period, had not undergone other behavioral sleep treatments in the prior year, and scored higher than 7 on the ISI, a 7-item self-reported questionnaire characterizing insomnia severity over the 2 previous weeks (scores range from 0 to 28, with 0 to 7 indicating no clinically significant insomnia; 8 to 14, subthreshold insomnia; 15 to 21, moderately severe clinical insomnia; and 22 to 28, severe clinical insomnia). Individuals were not eligible if they reported untreated obstructive sleep apnea syndrome, narcolepsy, restless leg syndrome, periodic limb movement disorder, delayed sleep phase syndrome, or central apnea syndrome. Exclusion criteria included poorly controlled psychiatric disorders and a history of bipolar disorder, schizophrenia, alcohol or drug use disorder in the prior year, shift work, regular travel to a location more than 1 time zone away for 2 or more days, or current or expected pregnancy.

The study team screened participants for eligibility by telephone. The ISI (single survey) and Consensus Sleep Diary (CSD)^22^ (10 consecutive days) were sent via the REDCap system at baseline prior to randomization. If participants completed 7 or more CSDs during the study run-in, they were randomized into the study. Participants also completed the ISI and CSDs via REDCap at the end of the study. A biostatistician designed randomization assignments in a 1:1 allocation ratio using random block sizes between 4 and 6. A sample size of 29 participants in each arm was required for an effect size of 0.65 and preintervention-postintervention correlation of 0.5 with 80% power. To account for 20% dropout, we recruited 76 participants (38 per arm) (Figure). Participants were enrolled between March 2022 and October 2023, and data collection was completed by December 2023.

**Figure.**
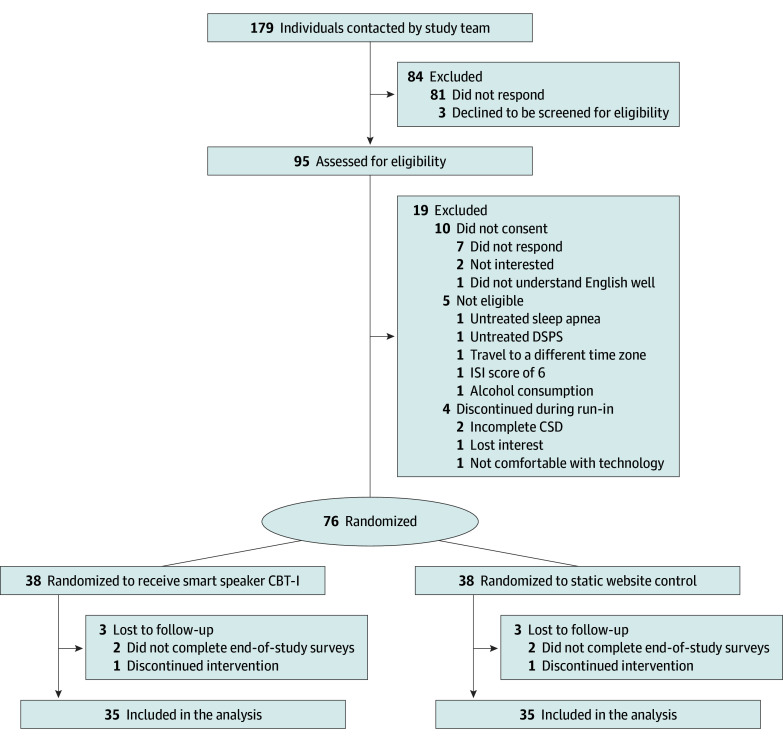
CONSORT Diagram for Recruitment and Retention CBT-I indicates cognitive behavioral therapy for insomnia; CSD, Consensus Sleep Diary; DSPS, delayed sleep phase syndrome; ISI, Insomnia Severity Index.

### Intervention

Participants assigned to the intervention received a smart speaker (Echo Dot, third generation [Amazon]) with a voice-activated program (Faster Asleep).^21^ At study intake, participants entered information on sleep concerns (eg, general sleep health and duration of insomnia), sleep patterns (bed and wake times, naps), sleep behaviors and sleep hygiene practice (eg, use of bed outside sleep times, caffeine intake), exercise, and use of sleep aids on the accompanying smartphone application (a smartphone was provided to participants). After study intake, all content was delivered by voice interaction. Participants activated the program by saying “Echo, launch Faster Asleep.” After collecting sleep diaries, the program provided education on fundamental CBT-I components, including sleep restriction, schedule modifications, stimulus control, and sleep hygiene. Only general education could be read on the app; participants could not complete sleep diaries or receive tailored feedback, including sleep restriction, without voice activation. After completion of nightly questionnaires, the program offered relaxation content. Recommendations based on CBT-I were tailored to individuals’ responses to the intake data and algorithms incorporating daily responses on sleep diaries.

### Control

Control group participants received access to a website developed by the research team with information about CBT-I, sleep, and cancer survivorship. Participants could engage with the website as desired within a 6-week period. There was no tailoring or interactive feedback.

### Outcome Measures

The primary outcome was change in scores on the ISI. A decrease of 7 points is considered moderate improvement and 8 points is marked improvement.^23^ All participants who completed the ISI at baseline were included in mixed models; additional analyses were performed with the participants who completed both beginning and end-of-study forms.

Secondary outcomes were calculated using results from the CSD at baseline and end of study. Three members of the study team (C.M.S., D.L., and H.A.) performed quality control of sleep diary data. Observations exceeding a 2-hour deviation from typical sleep or wake times or those with unexplained logical inconsistencies in the sleep diary were reviewed by a board-certified sleep specialist (D.L.), who determined exclusion for values that indicated incorrect diary completion. Additionally, observations recorded during travel across more than 1 time zone were omitted, and when time in bed and time to sleep were reversed, values were switched. After quality control, between 6 and 10 observations per participant at baseline and 2 and 10 observations per participant at end of study were used in mixed models. Sleep diary data yielded the following variables: sleep quality, wake after sleep onset, sleep onset latency, total sleep time, and sleep efficiency. Participants were asked to rate sleep quality on a 5-point Likert scale from very poor to very good, and sleep diary responses were averaged to generate a single score per participant. Total sleep time was calculated by subtracting sleep onset latency and wake after sleep onset from sleep duration (time to sleep to time awake). Time in bed was computed from the period between going in bed and getting out of bed, and sleep efficiency was derived from the ratio of total sleep time to time in bed.

At baseline and the end of study, participants also completed the Patient-Reported Outcomes Measurement Information System (PROMIS) 29-item scale in REDCap. Seven domains include 4 items each on a 5-point Likert scale: physical function, anxiety, depression, fatigue, ability to participate in social roles and activities, pain interference, and sleep disturbance. A single pain-intensity item was also included.^24,25^ We also administered the System Usability Scale (SUS)^26,27^ and evaluated participant satisfaction with the smart speaker program by asking participants whether they would recommend it to friends or family. To assess acceptability and appropriateness, we used an 8-item measure with Likert scale responses from 1 to 5, where higher scores were better.^28^

### Covariates

Participants self-reported demographic and medical history data, including age, sex, cancer stage at diagnosis, years since diagnosis, years with insomnia, height, weight, race, ethnicity, marital status, employment status, educational level, and sleep aid use. Race and ethnicity were collected per federal reporting standards; categories included Asian, Black or African American (hereafter, *Black*), White, multiracial, and Hispanic or Latinx. We purposively recruited from a hospital serving a large proportion of Black individuals with a history of cancer, as many previous studies on CBT-I for insomnia have included overwhelmingly White populations yet Black patients with cancer have worse outcomes than their White counterparts.^29^ We asked all participants about motivation to complete the study and expectation to see benefit on a Likert scale of 1 to 5, with 1 being highly motivated or expecting significant improvement and 5 indicating not at all motivated or expecting very little improvement. We also collected the Morningness-Eveningness Questionnaire short form, which includes 5 items that distinguish between morning and evening types.^30^ Participants received $75 and an Echo Dot for completing end-of-study questionnaires.

### Statistical Analysis

Baseline data were summarized using percentages, means with SDs, and medians with IQRs. We used D’Agostino-Pearson tests for normality. Fisher exact test, Student *t* test, and Kruskal-Wallis rank sum test were performed as appropriate to compare differences between groups. We used linear mixed models for analyses of all participants and Cohen *d* estimates among those who completed the study, with the corresponding percentile to evaluate effect size. Given the multiple measurements per person for sleep diary data, we used linear mixed models fit by restricted maximum likelihood. Regressor time points, arms, and an interaction term were included in the model. Estimated marginal means were calculated to present the start, end, and start-end contrast of the trial arms. Analyses were conducted in R, version 4.3.1 (R Project for Statistical Computing). Two-sided *P* <.05 was considered significant.

## Results

A total of 95 breast cancer survivors were assessed for eligibility; 10 never consented, 5 did not meet eligibility criteria, and 4 discontinued during the run-in period (Figure). Among the 76 included participants, 38 were randomized to the intervention group and 38 to the control group. Mean (SD) age was 61.2 (9.3) years, and mean (SD) time from diagnosis was 9.6 (6.8) years (Table 1). Participants had a median body mass index (calculated as weight in kilograms divided by height in meters squared) of 25.8 (range, 17.8-48.3). Two participants (2.6%) identified as Asian, 16 (21.1%) as Black, 2 (2.6%) as Hispanic or Latinx, 53 (69.7%) as White, and 3 (3.9%) as multiracial. Over half of participants were married or partnered (49 [64.5%]). With regard to employment, 29 (38.2%) worked full-time, 11 (14.5%) worked part-time, 31 (40.8%) were retired, and 5 (6.6%) were unemployed. Most participants had a college or graduate-level degree (66 [86.8%]). Participants had experienced insomnia for a mean (SD) of 10.3 (9.4) years. There was a varying distribution of breast cancer stage at diagnosis, with 2 participants (2.6%) at stage 0; 33 (43.4%), stage 1; 29 (38.2%), stage 2; 8 (10.5%), stage 3; and 4 (5.3%), stage 4. We compared the 70 people who completed the intervention (92.1%) with the 6 (7.9%) who did not and found no statistically significant differences except by race, by which the proportion of individuals who dropped out was higher among multiracial participants (1 of 3 [33.3%]) and Black participants (3 of 16 [18.8%]) compared with White participants (1 of 53 [1.9%]) (*P* = .03).

**Table 1.** Baseline Participant Demographics

Characteristic	Participants^a^		
	Overall (N = 76)	Intervention (n = 38)	Control (n = 38)
Age, mean (SD), y	61.2 (9.3)	62.6 (9.8)	59.8 (8.7)
Cancer stage at diagnosis			
0	2 (2.6)	0	2 (5.3)
1	33 (43.4)	21 (55.3)	12 (31.6)
2	29 (38.2)	12 (31.6)	17 (44.7)
3	8 (10.5)	3 (7.9)	5 (13.2)
4	4 (5.3)	2 (5.3)	2 (5.3)
Time since diagnosis, mean (SD), y	9.6 (6.8)	9.1 (7.2)	10.2 (6.3)
Time with insomnia, mean (SD), y	10.3 (9.4)	10.7 (10.9)	9.9 (7.6)
BMI, median (IQR)	25.8 (23.6-29.2)	25.9 (24.1-29.7)	25.6 (22.9-29.2)
Race			
Asian	2 (2.6)	0	2 (5.3)
Black or African American	16 (21.1)	7 (18.4)	9 (23.7)
White	53 (69.7)	28 (73.7)	25 (65.8)
Multiracial	3 (3.9)	2 (5.3)	1 (2.6)
Prefer not to answer	2 (2.6)	1 (2.6)	1 (2.6)
Ethnicity			
Hispanic or Latinx	2 (2.6)	2 (5.3)	0
Not Hispanic or Latinx	74 (97.4)	36 (94.7)	38 (100)
Marital status			
Single	13 (17.1)	6 (15.8)	7 (18.4)
Married or partnered	49 (64.5)	25 (65.8)	24 (63.2)
Divorced or separated	10 (13.2)	5 (13.2)	5 (13.2)
Widowed	4 (5.3)	2 (5.3)	2 (5.3)
Employment status			
Full-time	29 (38.2)	13 (34.2)	16 (42.1)
Part-time	11 (14.5)	4 (10.5)	7 (18.4)
Retired	31 (40.8)	16 (42.1)	15 (39.5)
Unemployed	5 (6.6)	5 (13.2)	0
Educational level			
High school, GED, or some college	10 (13.2)	6 (15.8)	4 (10.5)
College degree	30 (39.5)	14 (36.8)	16 (42.1)
Graduate degree	36 (47.4)	18 (47.4)	18 (47.4)
Sleep aids			
Herbal supplements	13 (17.1)	7 (18.4)	6 (15.8)
Cannabis	7 (9.2)	3 (7.9)	4 (10.5)
Over-the-counter medication	23 (30.3)	10 (26.3)	13 (34.2)
Prescription medication	13 (17.1)	2 (5.3)	11 (28.9)
Expected study outcomes, mean (SD)			
Expected to improve^b^	2.7 (1.0)	2.6 (0.9)	2.8 (1.0)
Motivated to complete^c^	1.6 (1.2)	1.5 (1.1)	1.7 (1.2)
Morningness-Eveningness score, mean (SD)^d^	15.0 (4.2)	15.0 (3.6)	15.0 (4.7)

Abbreviations: BMI, body mass index (calculated as weight in kilograms divided by height in meters squared); GED, General Educational Development.

^a^ Data are presented as number (percentage) of participants unless otherwise indicated.

^b^ Score range 1 to 5, with 1 indicating significant improvement and 5, very little improvement.

^c^ Score range 1 to 5, with 1 indicating extremely motivated and 5, not at all motivated.

^d^ Score range 4 to 25, with lower scores indicating more of an evening tendency and higher scores, a morning tendency.

### Primary Outcome

We found a significantly greater reduction in ISI score among intervention participants (mean [SD] score at baseline, 16.0 [3.5]; 6-week follow up, 7.4 [3.5]) compared with control participants (mean [SD] score at baseline, 15.4 [4.4]; 6-week follow-up, 12.7 [4.9]) (*P* < .001) (Table 2). The mean (SD) of the difference in ISI scores at the patient level was −8.4 (4.7) points in the intervention group compared with −2.6 (3.5) in the control group (*P* < .001); the estimated mean difference among those who completed the study was 5.83 (95% CI, 3.84-7.81), and Cohen *d* was 1.41 (95% CI, 0.87-1.94). The linear mixed model with all participants yielded the same contrast estimate. In further analysis stratified by median baseline ISI score, there was a mean (SD) change of −10.8 (4.3) for intervention participants and −3.8 (4.2) for control participants among those with a baseline ISI score higher than 15 compared with mean (SD) changes of −6.4 (4.1) and −1.6 (2.5) for intervention and control participants, respectively, for those with a baseline ISI score of 15 or lower. At the 6-week follow-up, 31 of the 35 individuals in the control arm (88.6%) had a worse score than the typical participant in the intervention group. To assess clinically relevant remission, the percentage of participants with an ISI score of 14 or lower (subclinical insomnia) after the intervention was considered; 94.3% (33 of 35 participants) in the intervention group reached the threshold, while 71.4% (25 of 35 participants) in the control group reached the threshold. In assessment of the percentage of participants who reached a score of 7 or lower (no clinically significant insomnia), 51.4% (18 participants) in the intervention arm met the criteria for remission compared with 11.4% (4 participants) in the control group.

**Table 2.** Change in ISI Scores From Baseline to End of Study

	ISI score, mean (SD)		*P* value	Difference in mean (95% CI)	Cohen *d* estimate (percentile) [95% CI]
	Intervention	Control			
**Overall** ^a^					
Baseline	16.0 (3.5)	15.4 (4.4)	.53	0.58 (−1.25 to 2.41)	0.14 (55.6) [−0.31 to 0.60]
6 wk	7.4 (3.5)	12.7 (5.9)	<.001	5.26 (3.23 to 7.28)	1.24 (89.3) [0.72 to 1.76]
Difference	−8.4 (4.7)	−2.6 (3.5)	<.001	5.83 (3.84 to 7.81)	1.41 (92.1) [0.87 to 1.94]
**ISI score >15^b^**					
Baseline	18.6 (2.8)	19.3 (2.6)	.47	0.65 (−1.16 to 2.45)	0.24 (59.4) [−0.43 to 0.91]
6 wk	7.9 (3.7)	15.2 (5.3)	<.001	7.25 (3.93 to 10.57)	1.58 (94.3) [0.76 to 2.41]
Difference	−10.8 (4.3)	−3.8 (4.2)	<.001	7.06 (3.99 to 10.13)	1.66 (95.2) [0.82 to 2.50]
**ISI score ≤15^c^**					
Baseline	13.3 (1.7)	11.9 (2.2)	.03	−1.42 (−2.69 to −0.14)	0.72 (76.4) [0.05 to 1.39]
6 wk	6.9 (3.4)	10.5 (3.2)	.002	3.58 (1.40 to 5.76)	1.08 (86.0) [0.38 to 1.79]
Difference	−6.4 (4.1)	−1.6 (2.5)	<.001	4.79 (2.54 to 7.04)	1.41 (92.1) [0.68 to 2.15]

Abbreviation: ISI, Insomnia Severity Index.

^a^ There were 38 participants in each arm at baseline and 35 in each arm at 6 weeks.

^b^ Of 37 participants, 19 were in the intervention arm and 18 in the control arm at baseline. At 6 weeks, there were 16 in the intervention arm and 16 in the control arm.

^c^ Of 39 participants, 19 were in the intervention arm and 20 in the control arm at baseline. At 6 weeks, there were 16 in the intervention arm and 19 in the control arm.

### Secondary Outcomes

Sleep diary data showed improvements from baseline to end of study across multiple domains (Table 3). We observed statistically significant improvements in estimated marginal means for sleep quality (0.56; 95% CI, 0.39-0.74), wake after sleep onset (9.54 minutes; 95% CI, 1.93-17.10 minutes), sleep onset latency (8.32 minutes; 95% CI, 1.91-14.70 minutes), time in bed (0.42 hours; 95% CI, 0.16-0.69 hours), and sleep efficiency (−0.04%; 95% CI, −0.07% to −0.01%) in the intervention group compared with the control group but no statistically significant difference for total sleep time (0.01 hours; 95% CI, −0.27 to 0.29 hours).

**Table 3.** Estimated Marginal Means for Sleep Diary Variables Comparing the Intervention and Control Arms and Linear Mixed Model Results^**a**^

Variable	Intervention, mean (SE) [95% CI]	Control, mean (SE) [95% CI]	Difference between arms, mean (SE) [95% CI]^b^	*P* value
**Sleep quality**				
Baseline	3.10 (0.08) [2.94-3.26]	3.12 (0.08) [2.96-3.28]	0.56 (0.09) [0.39 to 0.74]	<.001
6 wk	2.44 (0.08) [2.28-2.61]	3.03 (0.08) [2.86-3.19]		
**Wake after sleep onset, min**				
Baseline	45.50 (4.03) [37.50-53.50]	35.70 (4.02) [27.80-43.70]	9.54 (3.88) [1.93 to 17.10]	.01
6 wk	26.90 (4.08) [18.80-35.00]	26.60 (4.14) [18.40-34.90]		
**Sleep onset latency, min**				
Baseline	30.60 (2.91) [24.80-36.30]	35.00 (2.91) [29.30-40.80]	8.32 (3.27) [1.91 to 14.70]	.01
6 wk	16.80 (2.96) [10.90-22.70]	29.60 (3.02) [23.60-35.60]		
**Total sleep time, h**				
Baseline	6.46 (0.18) [6.11-6.81]	6.62 (0.18) [6.27-6.97]	0.01 (0.15) [−0.27 to 0.29]	.95
6 weeks	6.71 (0.18) [6.36-7.07]	6.89 (0.18) [6.53-7.24]		
**Time in bed, h**				
Baseline	8.92 (0.19) [8.54-9.29]	8.85 (0.19) [8.47-9.23]	0.42 (0.14) [0.16 to 0.69]	.002
6 wk	8.30 (0.19) [7.92-8.68]	8.66 (0.19) [8.27-9.04]		
**Sleep efficiency, %**				
Baseline	0.73 (0.02) [0.70-0.76]	0.75 (0.02) [0.72-0.78]	−0.04 (0.01) [−0.07 to −0.01]	.002
6 wk	0.82 (0.02) [0.79-0.84]	0.80 (0.01) [0.77-0.83]		

^a^ Models were adjusted for regressor time points, arms, and the interaction term.

^b^ Differences calculated as values at 6 weeks minus at baseline.

Comparing quality of life from baseline to end of study by group, there was no statistically significant difference for ability to participate in social roles or activities, anxiety, depression, pain interference, or physical function (Table 4). A statistically significant improvement in sleep disturbance was observed, with the intervention group showing a mean (SD) score change of −10.7 (7.0) compared with a change of −1.9 (4.7) in the control group, a difference of 8.79 (95% CI, 5.96-11.61). The PROMIS scale T score is based on a population norm of 50 and an SD of 10, suggesting that the intervention group improved from 59.7 to 48.6, closing the gap to the population norm. Additionally, fatigue levels significantly improved in the intervention group (mean [SD] score change, −6.3 [7.6]) compared with the control group (−0.8 [7.9]), with a difference of 5.44 (95% CI, 1.78-9.11). Linear mixed model analysis supported these findings with contrast estimates for fatigue (5.39; 95% CI, 1.74-9.04) and sleep disturbance (8.62; 95% CI, 6.11-11.70).

**Table 4.** Changes in Quality-of-Life Measures Based on the PROMIS-29

	Mean (SD)		*P* value	Difference in mean (95% CI)	Cohen *d* estimate (percentile) [95% CI]
	Intervention	Control			
**Ability to participate in social roles**					
Baseline	50.7 (7.5)	52.1 (7.1)	.40	1.43 (−1.91 to 4.76)	0.20 (57.8) [−0.26 to 0.65]
6 wk	55.3 (7.6)	54.1 (8.7)	.55	−1.16 (−5.03 to 2.70)	0.14 (55.7) [−0.33 to 0.62]
Difference	4.2 (7.6)	1.5 (7.2)	.14	−2.64 (−6.14 to 0.86)	0.36 (64.0) [−0.12 to 0.83]
**Anxiety/fear**					
Baseline	53.0 (9.5)	54.2 (8.5)	.57	1.17 (−2.93 to 5.28)	0.13 (55.2) [−0.33 to 0.59]
6 wk	50.4 (9.0)	51.3 (9.1)	.68	0.90 (−3.37 to 5.18)	0.10 (54.0) [−0.37 to 0.57]
Difference	−1.9 (6.8)	−2.1 (7.5)	.92	−0.18 (−3.57 to 3.21)	0.02 (51.0) [−0.45 to 0.50]
**Depression/sadness**					
Baseline	47.2 (8.1)	48.1 (7.7)	.61	0.92 (−2.67 to 4.52)	0.12 (54.7) [−0.34 to 0.58]
6 wk	47.1 (7.2)	47.2 (6.8)	.95	0.10 (−3.21 to 3.40)	0.01 (50.6) [−0.46 to 0.49]
Difference	−1.9 (6.8)	−2.1 (7.5)	.92	−0.18 (−3.57 to 3.21)	0.02 (51.0) [−0.45 to 0.50]
**Fatigue**					
Baseline	55.8 (7.2)	54.0 (8.5)	.32	−1.81 (−5.42 to 1.79)	0.23 (59.1) [−0.23 to 0.69]
6 wk	48.9 (6.8)	52.4 (8.5)	.06	3.53 (−0.13 to 7.18)	0.46 (67.7) [−0.02 to 0.94]
Difference	−6.3 (7.6)	−0.8 (7.9)	.004	5.44 (1.78 to 9.11)	0.70 (75.9) [0.22 to 1.19]
**Pain interference**					
Baseline	55.4 (8.3)	50.2 (7.6)	.006	−5.19 (−8.82 to −1.57)	0.66 (74.4) [0.19 to 1.12]
6 wk	52.9 (7.9)	49.5 (8.4)	.09	−3.32 (−7.19 to 0.56)	0.41 (65.8) [−0.07 to 0.88]
Difference	−1.6 (6.1)	−0.5 (6.6)	.46	1.13 (−1.87 to 4.13)	0.18 (57.1) [−0.30 to 0.65]
**Physical function**					
Baseline	49.7 (7.6)	51.0 (6.4)	.39	1.38 (−1.83 to 4.60)	0.20 (57.8) [−0.26 to 0.66]
6 wk	50.9 (7.1)	50.6 (7.2)	.86	−0.30 (−3.70 to 3.09)	0.04 (51.7) [−0.43 to 0.52]
Difference	1.0 (5.3)	−0.3 (5.1)	.29	−1.33 (−3.81 to 1.14)	0.26 (60.1) [−0.22 to 0.73]
**Sleep disturbance**					
Baseline	59.7 (5.0)	58.2 (6.0)	.27	−1.42 (−3.94 to 1.10)	0.26 (60.2) [−0.20 to 0.72]
6 wk	48.6 (6.6)	56.2 (5.3)	<.001	7.61 (4.78 to 10.45)	1.27 (89.8) [0.75 to 1.79]
Difference	−10.7 (7.0)	−1.9 (4.7)	<.001	8.79 (5.96 to 11.61)	1.47 (92.9) [0.93 to 2.00]

Abbreviation: PROMIS-29, Patient-Reported Outcomes Measurement Information System 29-item scale.

Intervention group participants reported a mean (SD) SUS score of 72.2 (16.6; range, 22.5-100), exceeding the average rating of 68. The mean (SD) acceptability score was 3.81 (0.9) of 5. Similarly, the mean (SD) appropriateness score was 3.85 (0.9) of 5. Furthermore, 30 of 35 intervention participants (85.7%) strongly agreed or agreed that they were satisfied with the program, and 30 of 35 (85.7%) reported that they would recommend the smart speaker program to friends or family. For the control group, the mean (SD) SUS score was 50.79 (15.9; range, 15.0-92.5). The mean (SD) acceptability score was 2.93 (0.9) of 5. The mean (SD) appropriateness score was 2.89 (0.9) of 5. Among the control group, 11 of 35 participants (31.4%) expressed satisfaction, while 14 of 35 participants (40.0%) indicated they would recommend the program to others.

### Engagement

The 35 participants who completed the study in the intervention arm completed between 15 and 42 days of daily sleep diaries (mean [SD], 39 [10.22] days) and education content (mean [SD], 39 [10.33] days), and 25 (71.4%) completed all diaries and education during the program; this information was used to provide sleep restriction guidance daily to achieve greater than 85% sleep efficiency. As nightly questionnaires were not required for new recommendations to generate, nighttime log completion ranged from 1 to 41 days (mean [SD], 31 [12.62] days); 5 participants (14.3%) completed all nightly questionnaires. Among the 35 in the control arm who completed the study, 29 (82.9%) engaged with the website at least once. The mean (SD) number of website visits was 5.55 (5.23; range, 1-26).

## Discussion

In this randomized clinical trial, participants using the smart speaker program showed marked improvements in insomnia symptoms compared with the control group.^23^ These findings support a novel method for addressing insomnia symptoms that presents new avenues for scalability and engagement.^31^

Our findings are similar to earlier studies using digital CBT-I programs such as SHUTi and I-Sleep, which have demonstrated similar improvements in insomnia severity (Cohen *d* ranging from 1.33 to 2.32) and sleep diary measures, although some previous studies showed a stronger effect in both shorter- and longer-term outcomes.^32,33,34,35,36^ The null results for total sleep time are similar to previous digital CBT-I findings^33^; this is not surprising, as CBT-I is focused on sleep initiation and continuity rather than total sleep time. Some of the participants in previous studies reported a slightly higher starting ISI score (mean, 17-18) than individuals in our study (mean, 15-16), which might explain a stronger effect size. Still, our findings suggest a robust effect on insomnia symptoms. A previous study also explored the effect of digital CBT-I on quality of life.^37^ Other studies have reported small to moderate improvements in fatigue (Cohen *d*, 0.24-0.44).^35,38,39^ While only 2 quality-of-life domains showed a difference by arm in our study, many of the quality-of-life domains may be less likely to change with improvements in insomnia symptoms. Our finding of changes in fatigue and sleep disturbance are expected, as these variables are directly related to decrements in sleep quality associated with insomnia. The smart speaker program was largely deemed user friendly, acceptable, and appropriate for its intended use. However, the variation in SUS scores, especially, suggests room for improvement. Technological issues with the study hotspots provided may have contributed to lower usability scores.

### Strengths and Limitations

Study strengths include an innovative approach to administer CBT-I daily at home. While previous research^40,41^ has successfully delivered digital CBT-I, this study is unique in providing daily interaction and feedback to increase sleep efficiency and adherence to sleep recommendations. In contrast, most other CBT-I programs work on a weekly cadence. In addition to the daily interaction, this program embeds in the home and allows for interaction intended to mimic in-person therapy.

This study also has limitations. Despite efforts to recruit from a hospital with a large Black population and lower socioeconomic status, 69.7% of the study’s participants were White and 86.8% had completed college or graduate school. Consequently, our findings may be less generalizable to other populations. Still, enrollment of Black individuals (21.1%) was higher than in other landmark digital CBT-I studies, in which the percentage of Black participants was 7% or less^33,42^ or the population was all White.^14,36^ Additionally, we were unable to assess impact beyond 6 weeks. Moreover, reliance on self-reported sleep measures introduces the possibility of bias due to inaccurate recall. However, prior research has shown sleep diaries to be reliable and valid,^33^ and our technology for sleep data collection ensured that data from previous days could not be entered, reducing recall bias. Additional analyses of secondary data suggested that participants with more missing diaries had a smaller change in sleep quality, suggesting bias in reporting but also that effect size estimates may be conservative. Still, in future studies, stratified randomization by baseline sleep diary completion may be prudent to reduce bias in missingness.

## Conclusions

Our study findings support the efficacy of a daily, in-home, voice-interactive CBT-I program among breast cancer survivors. Cancer centers may consider programs such as this to reach more breast cancer survivors with insomnia. Future studies should explore potential for scaling in-home sleep programs and increasing application of artificial intelligence and should compare engagement and noninferiority with other effective CBT-I programs.

## Notes

Supplement 1.
Trial Protocol

Supplement 2.
Data Sharing Statement
