# Supplementary material for: Voice-Activated Cognitive Behavioral Therapy for Insomnia: A Randomized Clinical Trial
Source: JAMA Netw Open. 2024 Sep 24;7(9):e2435011. doi: 10.1001/jamanetworkopen.2024.35011 (PMC11423177; doi:10.1001/jamanetworkopen.2024.35011)
Supplement: Supplement 1. — Trial Protocol [file jamanetwopen-e2435011-s001.pdf]

INSTRUCTIONS:

PROTOCOL TITLE:

Testing mHealth Delivery of Cognitive Behavioral Therapy for Insomnia to Breast Cancer Survivors

PRINCIPAL INVESTIGATOR:

Hannah Arem
Healthcare Delivery Research
202-893-2430
Hannah.Arem@medstar.net

VERSION NUMBER/DATE:

Version 1.0 September 8, 2021

REVISION HISTORY

Revision #	Version Date	Summary of Changes	Consent Change?

Table of Contents

1.0 Study Summary 3
2.0 Objectives* 5
3.0 Background* 5
4.0 Study Endpoints* 8
5.0 Study Intervention/Investigational Agent 9
6.0 Procedures Involved* 10
7.0 Data and Specimen Banking* 11
8.0 Sharing of Results with Subjects* 12
9.0 Study Timelines* 13
10.0 Inclusion and Exclusion Criteria* 13
11.0 Vulnerable Populations* 14
12.0 Local Number of Subjects 14
13.0 Recruitment Methods 14
14.0 Withdrawal of Subjects* 15
15.0 Risks to Subjects* 17
16.0 Potential Benefits to Subjects* 17
17.0 Data Management* and Confidentiality 17
18.0 Provisions to Monitor the Data to Ensure the Safety of Subjects* 21
19.0 Provisions to Protect the Privacy Interests of Subjects 24
20.0 Compensation for Research-Related Injury 25
21.0 Economic Burden to Subjects 25
22.0 Consent Process 25
23.0 Process to Document Consent in Writing 27
24.0 Setting 28
25.0 Resources Available 28
26.0 Multi-Site Research* 29

GHUCCTS Questions

GHUCCTS is the Georgetown-Howard Universities Center for Clinical and Translational Science.

Is this study a GHUCCTS Study? ____Yes __X__No

Is the project being sponsored or funded by GHUCCTS? ____Yes __X__No

Does the project utilize GHUCCTS services or facilities? ____Yes _X___No
(e.g., is the study conducted on the Clinical Research Unit (CRU), is the study supported by a GHUCCTS biostatistician, etc.)

1.0 STUDY SUMMARY

<table>
<tr><td>Study Title</td><td>Testing mHealth Delivery of Cognitive Behavioral Therapy for Insomnia to Breast Cancer Survivors</td></tr>
<tr><td>Study Design</td><td>Randomized controlled trial</td></tr>
<tr><td>Primary Objective</td><td>To determine whether the smart speaker group will have significantly greater reductions in insomnia severity compared to the website-basd group as recorded by the Insomnia Severity Index (ISI).</td></tr>
<tr><td>Secondary Objective(s)</td><td>Secondary Objective: To measure differences between intervention and control arms for ISI sub-scales</td></tr>
<tr><td>Research Intervention(s)/ Investigational Agent(s)</td><td>In the intervention arm participants will receive an Amazon Dot device with a pre-loaded Sleep Helper program, using an account created by Media Rez (study prime) so Amazon does not have access to participant names (for patient privacy purposes). Participants will be asked to engage with the program daily, either using the voice-activated speakers or an accompanying smart-phone app. The study duration is six-weeks and is completed individually using fully automated interaction to deliver components of cognitive behavioral therapy for insomnia.</td></tr>
<tr><td>IND/IDE #</td><td>N/A</td></tr>
<tr><td>Study Population</td><td>We will recruit female breast cancer survivors over age 18. Women must have been diagnosed with stage I-III breast cancer or stage IV with high functioning (Eastern Cooperative Oncology Group status 0-1). Curative treatment (not adjuvant) must have been completed >3 months prior to enrollment.</td></tr>
<tr><td>Sample Size</td><td>n=76 breast cancer survivors</td></tr>
<tr><td>Study Duration for individual participants</td><td>Individuals will fill out sleep and medical history forms at baseline and will be randomized to a six-week protocol.</td></tr>
<tr><td>Study Specific Abbreviations/ Definitions</td><td>
<table>
<tr><td>ACP</td><td>American College of Physicians</td></tr>
<tr><td>AE</td><td>Adverse Event</td></tr>
<tr><td>AI</td><td>Artificial Intelligence</td></tr>
<tr><td>ANCOVA</td><td>Analysis of Covariance</td></tr>
<tr><td>AWS</td><td>Amazon Web Services</td></tr>
<tr><td>BCS</td><td>Breast Cancer Survivors</td></tr>
<tr><td>CBT-I</td><td>Cognitive Behavioral Therapy for Insomnia</td></tr>
<tr><td>CFR</td><td>Code of Federal Regulations</td></tr>
<tr><td>CMP</td><td>Clinical Monitoring Plan</td></tr>
<tr><td>COC</td><td>Certificate of Confidentiality</td></tr>
<tr><td>CONSORT</td><td>Consolidated Standards of Reporting Trials</td></tr>
<tr><td>CRF</td><td>Case Report Form</td></tr>
<tr><td>CSD</td><td>Consensus Sleep Diary</td></tr>
<tr><td>DCC</td><td>Data Coordinating Center</td></tr>
<tr><td>DHHS</td><td>Department of Health and Human</td></tr>
</table>
</td></tr>
</table>

	Services
DSMB	Data Safety Monitoring Board
DRE	Disease-Related Event
EC	Ethics Committee
FDA	Food and Drug Administration
FFR	Federal Financial Report
GCP	Good Clinical Practice
GLP	Good Laboratory Practices
GMP	Good Manufacturing Practices
HIPAA	Health Insurance Portability and Accountability Act
IB	Investigator’s Brochure
ICH	International Council on Harmonisation
ICMJE	International Committee of Medical Journal Editors
IRB	Institutional Review Board
ISM	Independent Safety Monitor
ITT	Intention-To-Treat
MOP	Manual of Procedures
NCCN	National Comprehensive Cancer Network
NCT	National Clinical Trial
NIH	National Institutes of Health
NIH IC	NIH Institute or Center
OHRP	Office for Human Research Protections
PI	Principal Investigator
QA	Quality Assurance
QC	Quality Control
SAE	Serious Adverse Event
SAP	Statistical Analysis Plan
SMC	Safety Monitoring Committee
SOA	Schedule of Activities
SOP	Standard Operating Procedure
UP	Unanticipated Problem
US	United States

2.0 Objectives*

Specific Aim

To determine the impact of the voice-activated smart speaker Cognitive Behavioral Therapy for Insomnia (CBT-I) components on insomnia symptoms among breast cancer survivors. We will conduct a randomized controlled trial of n=76 breast cancer survivors to test the impact of a six-week in-home utilization of the CBT-I smart speaker program compared to static internet-based educational content on insomnia.

Objectives

OBJECTIVES
Primary
**To determine the impact of the voice-activated smart speaker CBT-I components on insomnia symptoms among breast cancer survivors using a randomized clinical trial.**
Secondary
**To determine differences in specific sub-scales of the ISI by intervention arm.**
Tertiary/Exploratory
**To determine whether there are differences in ISI change by frequency of engagement or satisfaction with the intervention.**

2.2

We hypothesize that our program will reduce insomnia measured by the insomnia severity index (ISI) to sub-clinical threshold levels overall (intent to treat analysis). We also will test whether those with higher adherence report greater improvement in insomnia symptoms.

3.0 Background*

Breast cancer survivors (BCS) numbered 3.6 million in the United States as of 2016; a number that is growing thanks to advances in early detection and treatment. However, long-term side effects such as persistent insomnia are experienced by ~30-50% of BCS and can have detrimental impacts on cardiometabolic and immune system health, neurobehavioral function, depression, fatigue, and quality of life, each of which in turn affect mortality. The National Comprehensive Cancer Network and the American College of Physicians recommend cognitive behavioral therapy for insomnia (CBT-I) as the first-line therapy for cancer survivors experiencing persistent sleep disruption. CBT-I targets physiologic, cognitive, behavioral, environmental, and social factors to improve sleep. CBT-I is a structured, systematic program which delivers better and safer outcomes than prescription sleep medications. However, identifying trained providers for CBT-I is challenging. Web-based therapy has shown efficacy, but published studies report dropout rates of up to 40%, which may be even higher in the real world. We propose developing and testing delivery of CBT-I treatment components using an in-home, voice activated smart speaker device. Our Phase I “Sleep Helper” prototype delivers important components of CBT-I interactively using a smartphone app and two paired smart speakers. As the system learns each user’s sources of insomnia, daily habits, and sleep schedule. it offers recommendations for changing sleep patterns based on CBT-I principles (going to bed an hour earlier, drinking less caffeine, leaving the

bedroom when you can't sleep, using a nighttime relaxation script, etc.). The system then monitors user progress for signs of improvement, building on what works and changing recommendations that are not working according to both subjective participant feedback and collected sleep data. Sleep Helper uses the core principles of CBTI to dynamically adapt each individual's treatment regimen, much like a sleep therapist would, and records it in a backend database for participant monitoring. The goal of this study is to develop a scalable, voice-activated smart speaker program to treat insomnia symptoms among BCS. Our long term-goal is to address insomnia among BCS and then adapt it for other high-risk populations.

Cognitive behavioral therapy was developed by Aaron Beck, MD in the 1970s with the goal of modifying depressed and anxious patient's negative thoughts and behavior patterns with sustainable improvements in mood, social and workplace functioning {Beck, 1970 #44}. Based on CBT principles, CBT-I was developed to address a complex set of symptoms, cognitions and behaviors that are specific to insomnia (Figure 1) {Yang, 2006 #52}. Therapists used validated tools to assess the contribution of psychophysiological and environmental factors that predispose a patient to insomnia, and used these to address specific symptoms including events that precipitate sleep disruption and negative attitudes about sleep that perpetuate insomnia, referred to as the "3 Ps" {Glovinsky, 2008 #47}. While CBT-I shares cognitive and stimulus control intervention strategies with CBT, the additional specialized components of CBT-I intervention include, sleep restriction, sleep education, hygiene training, and relaxation therapies.

Overview of CBT-I content, participant-directed enhancements, and Sleep Helper integration: In Phase I we built prototypes for core CBT-I techniques, tailored to BCS preferences as expressed during the focus groups. These techniques have been shown to be efficacious in improving sleep outcomes in a meta-analysis of internet delivered CBT-I {Zachariae, 2016 #135}, and will be developed to as closely as possible mimic therapist-delivered CBT-I.

1) Psychoeducation and cognitive restructuring. Cognitive behavioral therapy helps to shift common insomnia misconceptions and perceived poor sleep quality. For instance, participants learn that nighttime wakening is normal, and are coached to notice negative thoughts and replace them with positively framed messages. Phase I focus groups suggested that BCS appreciated educational content (e.g. circadian contribution and to sleep regulation and timing) and that they want short, targeted information as generally delivered during in-person CBT-I, with a "tell me more" prompt available if more information is desired. Phase I participants also expressed an interest in short quizzes after each section in order to enhance engagement. However, we will include options to turn off quizzes to meet individual preferences.

2) Sleep hygiene. Practical and general information about optimal sleep health, sleep hygiene and sleep/wake regulation are key CBT-I techniques to facilitate behavior change and set sleep health priorities. Phase I focus groups suggested that users were willing to receive sleep hygiene prompts from the Alexa program. Types of sleep hygiene recommendations that participants found acceptable and helpful included sleep schedule modifications, eliminating daytime napping, modified use of caffeine, nicotine, and alcohol, optimizing exercise and the bedroom environment, and curtailment of screen time use at night. Sleep Helper will use daily feedback on these behaviors to

make personalized change recommendations. Learning the importance of these behaviors has been found to significantly impact insomnia among cancer survivors {Recklitis, 2018 #63}. In addition to traditional CBT-I content, we will include information about breast cancer diagnosis, treatment and sequelae (e.g. hot flashes and frequent awakenings to use the bathroom) as it relates to insomnia, guided by input from Dr. Kaltman (co-I).

3) Relaxation exercises. Progressive muscle relaxation is a technique commonly used in CBT-I. Phase I participants expressed an interest in relaxation therapies with varying lengths and scripts to listen to in preparing for sleep. Participants will be able to sample a wide variety and keep the ones that work best for them by accepting or rejecting them through the program. We will include a smart speaker or pillow speaker/Bluetooth mask so as not to disturb a bed partner. The volume will slowly lower throughout playback until it is nearly silent by the end, to minimize the risk of re-awakening (participants can turn this feature off).

4) Stimulus control. Dissociating wakefulness and frustration lying in bed awake is a key component of CBT-I. The Echo is programmed so that the light ring maintains a faint glow that ends after 20 minutes. Thus, if the participant is awake when the light goes out, she knows to leave the bedroom without an audio prompt that could disturb a bed partner. A device in an alternate room will suggest that she read or listen to non-stimulating material, or will provide ideas for other calming activities initiated by voice commands (i.e., no use of screens). There will then be occasional prompts to query the degree of sleepiness. Users will only be prompted to return to the bedroom when sleepy.

5) Sleep restriction. Sleep restriction is a challenging aspect of CBT-I that directs individuals to use utmost discipline to reduce their sleep duration as a means of increasing their homeostatic sleep drive to ultimately consolidate and improve sleep continuity and quality. The amount of time spent in bed will be restricted to the user’s average total sleep time based on data from their automated sleep diary as determined by algorithms prescribed by CBT-I therapists Drs. Lewin and Zhou (Co-Investigators).

3.2 Describe any relevant preliminary data.

In Phase I of this project (R43CA232905) we assessed the feasibility and acceptability of the prototype for delivering CBT-I components using a smart speaker. After prototype demonstration, nearly 79% (11/14) of participants reported an interest in and perceived feasibility of using the virtual assistant to record sleep patterns. Approximately two-thirds of the participants thought lifestyle modification (9/14, 64%) and sleep restriction (9/14, 64%) would be feasible and were interested in this feature of the program (10/14, 71% and 9/14, 64%, respectively). Relaxation exercises were rated as interesting and feasible using the virtual assistant by 71% (10/14) of the participants. Usability was rated as better than average, and all women reported that they would recommend the program to friends and family.

We are currently conducting additional formative work with the prototype, and preliminary results from Aims 1 and 2 suggest participants found the delivery of CBT-I components using a smart speaker acceptable (Mean = 3.6/5 and 3.94/5 for Aim 1 and 2 respectively) and appropriate (Mean = 3.4/5 and 4.0/5 for Aim 1 and 2 respectively). System usability was rated above average (76% and 78% for Aims 1 and 2 respectively)

3.3

Insomnia is common in breast cancer survivors (BCS), affecting an estimated 30-50% of the 3.6 million breast cancer survivors in the US; insomnia has myriad psychological and medical sequelae that compound late and long-term effects of cancer diagnosis and treatment. Insomnia has detrimental health consequences for cardiometabolic and immune system health, neurobehavioral function, depression, fatigue, and quality of life. Compared with normal sleepers, those with insomnia had more physician-office visits, were hospitalized twice as often, and used more medication. Causes of insomnia may be multi-faceted, including cancer related physiological processes, iatrogenic effects of oncotherapies, menopause, and co-morbid mood disorders associated with cancer diagnosis and psychosocial and economic stressors. While pharmacotherapy for insomnia may rapidly address symptoms in the short-term, most have negative side effects, including decreasing efficacy over time, tolerance and dependence. Cognitive Behavioral Therapy for Insomnia (CBT-I) is considered to be the gold standard insomnia treatment and is recommended as first line treatment by the American College of Physicians (ACP) and the National Comprehensive Cancer Network (NCCN). Previous studies have shown efficacy of CBT-I in the BCS population. However, CBT-I trained practitioners are scarce (even in accredited sleep centers) and a standard regimen of multiple follow-up visits can inhibit completion, particularly among cancer survivors who are likely to have myriad health concerns as well as financial toxicity from disruptive cancer treatments. To address this, automated therapies delivered via the internet have been developed and shown to be effective. However, most of these programs are more didactic than interactive and require concerted effort on the part of the patient to adhere to the treatment protocol, resulting in declining participation over time. Where it is accessible, CBT-I is traditionally delivered in-person in 5-8 sessions, although recent studies have shown efficacy with a three session, brief treatment among cancer survivors. Still, these trials have shown dropout rates of 20-30% despite considerable effort from research staff to engage patients; it is likely that in real world settings non-adherence would be even higher.

Smart speaker devices may improve adherence as they embed a voice-activated program in the home and can provide real-time CBT-I patient-tailored strategies This study aims to further develop and test our voice-activated technology to deliver components of cognitive behavioral therapy for insomnia (CBT-I) to breast cancer survivors (BCS). CBT-I is a structured, systematic program which delivers better and safer outcomes than prescription sleep medications. Non-pharmacologic approaches target physiologic, cognitive, behavioral, environmental, and social factors to improve sleep. Insomnia has numerous health consequences, particularly for this high-risk population, and too few trained CBT-I therapists are available to offer treatment. Online programs suffer from high dropout rates and are not appropriate to use "just-in-time" before sleep (i.e. in the bedroom) due to stimulating screen exposures. Participants will interact with an internet-connected speaker to report sleep patterns and receive feedback, more closely mimicking the experience of an in-person provider than web-based programs.

4.0 Study Endpoints*

Completion of the six-week study, and relevant assessments- primarily the insomnia severity index. See below for the endpoints corresponding to the described objectives.

4.1

OBJECTIVES	ENDPOINTS
Primary	
**To determine the impact of the voice-activated smart speaker CBT-I components on insomnia symptoms among breast cancer survivors using a randomized clinical trial.**	We will collect data on the Insomnia Severity Index (ISI) total score pre- and post-intervention as our primary outcome. The ISI is a seven-item questionnaire with response categories from 0-4 (total score 0-28) asking about sleep patterns and specifically characterizing insomnia over the two weeks prior. The ISI defines 'no clinically significant' insomnia as a score of 0-7, 'sub-threshold' insomnia as a score of 8-14, 'moderate severity clinical' insomnia' a scores of 15-21, and 'severe clinical' insomnia as a score of 22-28. Our clinically relevant target for success is to achieve sub-threshold or better scores (≤14) among >80% of the intervention participants.
Secondary	
To determine differences in specific sub-scales of the ISI by intervention arm.	ISI subscales: severity of sleep onset, sleep maintenance and early morning awakening, sleep dissatisfaction, interference of sleep with daytime functioning, noticeability of sleep problems by others, and distress caused by sleep difficulties.
Tertiary/Exploratory	
To determine whether there are differences in ISI change by frequency of engagement or satisfaction with the intervention.	Among the intervention group we will also examine frequency of participant engagement and the internet intervention utility scale (IIUS), including participant satisfaction with the program, and whether these factors affect ISI change.

5.0 Study Intervention/Investigational Agent

Intervention Condition.

After randomization to the intervention arm participants will receive an Amazon Echo Dot with the Sleep Helper skill and will be asked to enroll either by giving responses to voice prompts for setup or entering baseline information via an accompanying smart phone application. Participants will receive written instructions for how to enroll. The MHRI research team will reach out to participants by email and/or phone after delivery confirmation to ensure that participants have enrolled and initiated the program. Participants will receive written instructions to complete morning, night, relaxation and educational modules daily. Participants will also be instructed on security settings that will be pre-set for maximal security.

Intake. At the start of the intervention participants will be provided with written instructions on setting the program up and asked to complete an intake form on background information, which can be completed via the smart phone app. Participants will be able to use a smart phone app to enter standard insomnia intake assessment

information that includes sleeping patterns, sleep hygiene practices (e.g. timing and frequency of caffeine use of screens, primary meals and exercise, etc.), whether they have a bed partner, and general background information for the study. The smart phone app will be linked with the smart speaker device so that information added in any format enters the same user backend database.

Participants will be encouraged to complete the morning and night modules daily, and to listen to relaxation and educational content or review written educational content on the accompanying smart phone app. The relaxation modules are prompted upon completion of the night module, although users are able to request a relaxation exercise at any point in time. Media Rez will maintain a technical support system through the app for user difficulties and will track all inquiries using de-identified participant ID numbers. App support rather than phone support will be utilized to maintain participant privacy so that phone numbers will not be revealed. The morning module collects sleep diary data from the preceding night and provides participant specific reminders based on participant data on naps, exercise and other daily activities. The evening module walks the user through the sleep schedule (e.g., recommended sleep bedtime schedule based on sleep restriction algorithm and participant specific bedtime routine guidance), questions about daily intake of caffeine and nicotine, napping, and exercise (data collection), and automated control of lighting to facilitate preparation for sleep. Educational programming includes short sleep education content and cognitive restructuring with the option to follow up with “tell me more” to get more information.

Control Condition. Controls will have access to a website with information about CBT-I and breast cancer survivorship and will be told to engage with the website as desired. The content will be drawn from the same script that is programmed into the Alexa device.

6.0 Procedures Involved*

Type of study design. We will conduct a six-week, individual randomized controlled trial with two arms. While recruitment is being conducted at various locations, the intervention is delivered remotely and all data collection and analyses will be conducted through Medstar Health Research Institute.

Individuals who express an interest in the study will be screened for eligibility by phone. Questionnaires will be completed in REDCap. Study staff will download screening results and calculate ISI scores. Participants will be notified of eligibility by phone or email within 3 business days of completing the eligibility screening.

If the participant is eligible and wishes to proceed with study enrollment, she will receive a personalized REDCap link by email with baseline forms. Study staff will be available to administer the forms by phone or to send hard copies by mail if the individual prefers. Prior to randomization we will collect information on basic demographics (e.g. age, body mass index, education, income, marital status), medical history (e.g. comorbidities, time since treatment, concurrent medication), and lifestyle habits (e.g. physical activity, smoking, alcohol consumption, diet) as well as our primary outcome of the Insomnia Severity Index (ISI). The ISI defines ‘no clinically significant’ insomnia as a score of 0-7, ‘sub-threshold’ insomnia as a score of 8-14, ‘moderate severity clinical’ insomnia’ a scores of 15-21, and ‘severe clinical’ insomnia as a score of 22-28.

Randomization. After screening for eligibility, participants will complete informed consent. Our biostatistics consultant will provide randomization assignments in a 1:1 allocation ratio using a block randomized design with random block sizes between 3-5.

During the study the Sleep Helper program will collect sleep diary data via voice in an ongoing manner. At the completion of the six-week intervention individuals will complete forms via a personalized REDCap link. If individuals complete >50% of the items on a scale mean substitution of missing values with respondent's average response on remaining scale items will be used.

This study will use Amazon Alexa Echo Dots. Participants will also receive a hotspot and a smartphone, so that they do not have to rely on personal wifi (thus further protecting their identity). These devices will be supplied to participants at no cost. We do not foresee any significant risk in using this device. This device is exempt from IDE regulation as it is in commercial distribution and is used in accordance with the indications in labeling.

Data collection. Baseline data before randomization will be collected from all participants using REDCap. We will have a separate link to a password protected recruitment document (with name and contact information) where unique study IDs will be assigned, thus further protecting the identity of participants. In the de-identified REDCap forms we will collect information on basic demographics (e.g. age, race/ethnicity, body mass index, education, marital status), medical history (e.g. comorbidities, time since treatment), and lifestyle habits (e.g. physical activity, alcohol consumption) as well as our primary outcome of the Insomnia Severity Index (ISI). We will also collect information on the PROMIS 29-item scale to see how co-occurring symptoms such as fatigue and anxiety are distributed by group. Additional information to be collected includes a morning/eveningness questionnaire to assess chronotype and participant expectations and motivations. All data will be self-reported.

Ongoing data collected through the Alexa app includes basic sleep information including time getting into bed, time attempting to go sleep, time awake during the night, time awake in the morning, time getting out of bed, number of awakenings, and quality of sleep. The ongoing data collected is used to inform treatment recommendations, but is not considered in the primary or secondary outcome analyses, which are based on the ISI.

7.0 Data and Specimen Banking*

No specimens are being collected for the purposes of this study.

MHRI will maintain appropriate medical and research records for this trial, in compliance with ICH GCP and regulatory and institutional requirements for the protection of confidentiality of participants. As part of participating in a NIH-sponsored or NIH-affiliated study, each site will permit authorized representatives of the NIH, sponsor, and regulatory agencies to examine (and when permitted by applicable law, to copy) clinical

records for the purposes of quality assurance reviews, audits, and evaluation of the study safety, progress, and data validity. Only trained study staff who need access to data for research purposes related to this project will have access to the data. Any data shared with Media Rez (software developer) will be deidentified.

Data Collection and Management Responsibilities

Data collection will be the responsibility of the MHRI trial staff at the site under Dr. Arem’s supervision. Dr. Arem will be responsible for ensuring the accuracy, completeness, legibility, and timeliness of the data reported, as well as for storing all informed consent documents and for managing any other identifiable information, including any audio recordings or transcripts.

Data will be entered into REDCap, a 21 CFR Part 11-compliant data capture system provided by MHRI. The data system includes password protection and internal quality checks, such as automatic range checks, to identify data that appear inconsistent, incomplete, or inaccurate.

While participants will be recruited from multiple sites, all records will be collected and stored by MHRI and only MHRI research staff who have a need will be able to see identifiable information.

Study Records Retention

Study documents will be retained for a minimum of 3 years after the last annual Federal Financial Report (FFR) is submitted.

8.0 Sharing of Results with Subjects*

Individuals can view their own ongoing data in the app at any point in time. Data collected through REDCap will not be shared with those outside the research team and will only be reported in the aggregate.

Publication and Data Sharing Policy

This study will be conducted in accordance with the following publication and data sharing policies and regulations:

National Institutes of Health (NIH) Public Access Policy, which ensures that the public has access to the published results of NIH funded research. It requires scientists to submit final peer-reviewed journal manuscripts that arise from NIH funds to the digital archive PubMed Central upon acceptance for publication.

This study will comply with the NIH Data Sharing Policy and Policy on the Dissemination of NIH-Funded Clinical Trial Information and the Clinical Trials Registration and Results Information Submission rule. As such, this trial will be registered at ClinicalTrials.gov, and results information from this trial will be submitted to ClinicalTrials.gov. In addition,

every attempt will be made to publish results in peer-reviewed journals. Data from this study may be requested from other researchers 1 year after the publication of the primary endpoint by contacting Dr. Hannah Arem at Medstar Health Research Institute.

All ongoing monitoring or self-reported data will be available to participants at any time during the study via the app. To maintain patient engagement, we will send electronic study updates twice a year on study progress (enrollment, process data, etc). At the study conclusion, we will send an electronic newsletter to all involved patients who do not opt out with summary findings from the study.

9.0 Study Timelines*

9.1

- Individuals will participate in the study for six weeks.
- We anticipate beginning recruitment for the study in late 2021, and enrolling the last participant by early 2023 (14 months total)
- We hope to complete the last data collection by Feb 2023, with analysis conducted by March 2023.

10.0 Inclusion and Exclusion Criteria*

The research coordinator will contact potentially eligible participants by phone to discuss the study and conduct screening informed consent. Those who agree to be screened for eligibility will complete the screening questionnaire with the research coordinator by phone.

Inclusion and Exclusion criteria

Inclusion Criteria

In order to be eligible to participate in this study, an individual must meet all of the following criteria:

1. Willingness to maintain a consistent dosing pattern if currently taking sleep medications or using cannabis for sleep
2. Females; Age 18+
3. Self-reported or documented diagnosis of breast cancer stage I-III or stage IV ECOG 0-1
4. Completed curative treatment (surgery, radiation, chemotherapy) > 3 months prior to enrollment [ongoing adjuvant therapy permitted]
5. Has not undergone other behavioral sleep treatment within the prior 12 months
6. Score greater than or equal to 8 on the Insomnia Severity Index
7. Able to understand and speak English

Exclusion Criteria

An individual who meets any of the following criteria will be excluded from participation in this study:

1. Diagnosed, untreated obstructive sleep apnea syndrome, narcolepsy, restless leg syndrome, periodic limb movement disorder, delayed sleep phase syndrome, central apnea
2. Bi-polar disorder, schizophrenia, initiation of psychological treatment within three months, alcohol or drug abuse in the prior year (Alcohol >2 drinks/day or consuming 5+ drinks in a single day in the prior month). (Moderate ADHD, depression and anxiety will not be exclusion criteria.)
3. Shift-work in the prior three months or anticipated during the study time
4. Planned regular travel out of time zone (>1 hour) during the study period.
5. Currently or planning to become pregnant during the study period

Indicate specifically whether you will include or exclude each of the following special populations:

- *Adults unable to consent- NO*
- *Individuals who are not yet adults (infants, children, teenagers)- NO*
- *Pregnant women- NO*
- *Prisoners- NO*

11.0 Vulnerable Populations*

N/A- We will not include adults who are unable to consent.
We will not include individuals who are not yet adults.
We will not include pregnant women.
We will not include prisoners.

12.0 Local Number of Subjects

12.1 We plan to enroll up to 76 patients in the study total, with about one third accrued locally.

We anticipate screening approximately 150 women in order to identify n=76 for the RCT.

13.0 Recruitment Methods

We will only screen women over age 18 who have been identified as breast cancer survivors who completed treatment and self-report disrupted sleep. Thus, we anticipate that main reasons for ineligibility will be due to other criteria such as planned travel out of the time zone, ineligible ISI score (7 or less), or other diagnosed untreated sleep disorder such as sleep apnea.

We are using multiple recruitment sources as detailed below to facilitate rapid enrollment.

a) Participants will be recruited from the Medstar Washington Hospital Center Cancer Institute, and Georgetown University's Lombardi Comprehensive Cancer

Center. The research coordinator will screen daily patient appointment schedules coming in for routine medical oncology visits after treatment completion to determine which patients might be eligible for the study. With consent from the treating physician, the research coordinator will call potentially eligible participants by phone or arrange to meet them in person if preferred to discuss the study, conduct screening, and informed consent. We will also distribute study brochures to MedStar Health breast cancer clinics to reach out to survivors who report insomnia. If recruitment is slow, we will reach out to the other MedStar cancer clinics to determine if clinic-based recruitment is feasible.

b) We will also reach out to local survivorship groups to distribute brochures, as was done in our previous studies of insomnia among BCS.
c) We will recruit from the Georgetown Lombardi Comprehensive Cancer Center's Survey, Recruitment, and Biospecimen Collection Share Resource (SRBSR), which includes nearly 2,300 cancer survivors who have consented to be contacted for future research.
d) We will recruit from the Susan Love Research Foundation Army of Women, a resource of 383,000 men and women interested in cancer research will also recruit participants. The recruitment flyer approved by Army of Women is attached to this application.
e) Additional participants will be recruited from the Dana-Farber Cancer Institute (DFCI)/Harvard Cancer Center using procedures proven successful in previous studies. The DFCI team will use medical records to identify potentially eligible patients from the breast oncology clinics, and recruit them at the time of a scheduled outpatient visit. This clinic-based recruiting strategy previously accrued 56 adult survivors in a 12-month period. The DFCI team will use medical records to determine clinical eligibility (cancer diagnosis, age, and time since treatment) and will approach women at their regular clinic visit whether they have trouble sleeping that began or got worse after their breast cancer diagnosis. Those who answer affirmatively will be referred to the MHRI team (via a private Box folder using a password protected spreadsheet) to administer the eligibility screening questionnaire. The DFCI team will not collect data for this study, and will not share medical records with the MHRI team.

All participants will receive a $75 incentive through electronic gift card at the completion of the 6-week post-intervention forms. At study completion (once all participants have completed the study), those who completed the post-intervention forms will also receive an Amazon Dot device (no account linked) that they can keep for personal use.

14.0 Withdrawal of Subjects*

DISCONTINUATION OF STUDY INTERVENTION/EXPERIMENTAL MANIPULATION

When a subject discontinues using the Sleep Helper skill but does not withdraw from the study, remaining study procedures will be completed as indicated by the study protocol.

The investigator or qualified designee will determine if any change in participant management is needed.

The data to be collected at the time of study intervention discontinuation will include the following:

- The reason(s) for discontinuing the participant from the intervention, and methods for determining the need to discontinue
- The participant will be asked to complete the post-interventions assessments, even though not participating in the intervention

PARTICIPANT DISCONTINUATION/WITHDRAWAL FROM THE STUDY

Participants are free to withdraw from participation in the study at any time upon request. An investigator may discontinue a participant from the study for the following reasons:

- Lost-to-follow up; unable to contact subject
- Any event or medical condition or situation occurs such that continued collection of follow-up study data would not be in the best interest of the participant or might require an additional treatment that would confound the interpretation of the study
- The participant meets an exclusion criterion (either newly developed or not previously recognized) that precludes further study participation

The reason for participant discontinuation or withdrawal from the study will be recorded on the Case Report Form (CRF). The CRF will include the subject ID, the date the subject left the study, who initiated withdrawal (subject vs PI), and reason for withdrawal. Subjects who sign the informed consent form and are randomized but do not receive the study intervention may be replaced. Subjects who sign the informed consent form, and are randomized and receive the study intervention, and subsequently withdraw, or are discontinued from the study, will not be replaced.

LOST TO FOLLOW-UP

A participant will be considered lost to follow-up if he or she fails to complete the post intervention forms and study staff are unable to contact the participant after 5 attempts.

The following actions must be taken if a participant fails to return post-intervention forms or equipment:

- Before a participant is deemed lost to follow-up, the investigator or designee will make every effort to regain contact with the participant (where possible, up to 5 telephone calls and/or emails, and if necessary, a mailed letter to the participant's last known mailing address or local equivalent methods. These contact attempts will be documented in the participant's study file.
- Should the participant continue to be unreachable, she will be considered to have withdrawn from the study with a primary reason of lost to follow-up.

15.0 Risks to Subjects*

We anticipate minimal risk to participants for participating in the study.

Known Potential Risks

Primary risks of the study include: a) embarrassment or discomfort with study questions; b) emotional, social, or physical harms resulting from the suggested behavioral modifications; c) inadvertent disclosure of data; d) Inadequacy of informed consent; or e) other risks that are unlikely to occur, but could. We discuss below methods to protect against these risks:

Embarrassment or discomfort with detailed behavioral questions or institutional structure can occur. This risk is minimized by ensuring that participants know they do not have to participate or may only answer the questions that they feel comfortable answering. We will refer patients to seek care from their regular medical doctor in the event the participant experiences any emotional or physical discomfort or will remind them that they can stop participation in the study at any point in time.

Data security:

- Data will be encrypted at all times, from the point of entry into RedCAP to the point of backup and to analysis. Data will be encrypted using standardized software (e.g., 128-bit, PGP).
- Data entered into RedCAP will be de-identified at all times. RedCAP results will be linked to a Survey ID number rather than names to minimize risk of inadvertent disclosure.
- Transfer of data will be overseen by Dr. Arem and conducted using password protected and encrypted files.
- Data will not be released to non-study staff at any time.
- Any audio-recorded data will be transcribed without identifiers and audio recordings will be destroyed after quality control or within five years.

16.0 Potential Benefits to Subjects*

Known Potential Benefits

The potential benefits from participation in this study include improvement in insomnia symptoms among randomized controlled trial intervention participants and in the longer term, dissemination of cognitive behavioral therapy for insomnia for breast cancer survivors. Knowledge gained from this project will be used to inform design of a commercial product to treat insomnia among cancer survivors. In view of the potential information to be gained, and how it will ultimately be able to contribute to new approaches to providing care to this survivor population, the minimal risks do not outweigh the potential for benefit.

17.0 Data Management* and Confidentiality

o STATISTICAL HYPOTHESES

- Primary Endpoint(s): Insomnia Severity Index

We hypothesize that, compared to participants who receive a control intervention, participants who receive Cognitive Behavioral Therapy for Insomnia via the Sleep Helper skill will have lower ISI scores at the end of the six-week intervention. Alternatively, our null hypothesis is that there will be no difference in the ISI score between groups at the end of the six-week intervention. Thus our primary efficacy outcome is based on the continuous ISI scale. We plan to also conduct a categorical analysis: we further hypothesize that a greater percentage of those in the intervention arm will have a clinically significant reduction in ISI score to <14, or sub-threshold insomnia, than in the control arm.

- Secondary Endpoint(s): ISI subscales

Our statistical objective is exploratory in that we are interested in whether we are seeing changes only in some but not all of the subscales in the ISI. We will evaluate the following ISI subscales: severity of sleep onset, sleep maintenance and early morning awakening, sleep dissatisfaction, interference of sleep with daytime functioning, noticeability of sleep problems by others, and distress caused by sleep difficulties.

- Exploratory Endpoint(s): We will analyze whether we see differences in ISI change by level of engagement and satisfaction with the intervention.

o SAMPLE SIZE DETERMINATION

We based our power calculations on the null hypothesis of no significant mean adjusted difference between groups post-intervention on a continuous total ISI score, after controlling for baseline score using Analysis of Covariance (ANCOVA). A systematic review and meta-analysis published in 2016 showed a large effect size of d=0.77 (95% CI 0.60-0.93) at post-treatment across seven randomized controlled trials {Johnson, 2016 #740}. A recent study of internet-based CBT-I among breast cancer survivors found an effect size of 1.17 where the outcome was the ISI {Zachariae, 2018 #69}.
We used Proc GLMPOWER in SAS Version 9.4 to calculate a series of required sample sizes with covariate-outcome correlations (0.5-0.8) with a desired power of 80% to detect effect sizes between .50 and .80 (medium to large). N values included in the table are for total sample size. Alpha was set at .05 (2-sided test).

**Effect size**	**Correlation 0.5**	**Correlation 0.6**	**Correlation 0.7**
**0.5**	N=98	N=84	N=68
**0.65**	N=58	N=50	N=40
**0.8**	N=40	N=34	N=28

Estimating a conservative effect size (based on prior literature) of 0.65 and a pre-post correlation of 0.5, we would need N=58 participants, or n=29 per arm. We will aim to recruit N=76 total (n=38 per arm) to allow for drop out of up to 30%.

o POPULATIONS FOR ANALYSES

For our primary and secondary analyses we will be using intent to treat. In tertiary analyses we will conduct analyses by adherence (median frequency of engagement).

○ STATISTICAL ANALYSES

General Approach

Aim 3. In the clinical trial analysis we will follow CONSORT guidelines to descriptively present means and frequencies of baseline characteristics in Table 1, and will follow guidelines in all analyses and reporting. We will consider $p<0.05$ as the level of significance and will use 2-tailed tests.

Analysis of the Primary Endpoint(s)

The ISI is a seven-item questionnaire with response categories from 0-4 (total score 0-28) asking about sleep patterns and specifically characterizing insomnia over the two weeks prior. Our primary analysis endpoint will use this continuous ISI score.

We will test for assumptions of linearity of regression, homogeneity of error variances, independence of error terms, normality of error terms, and homogeneity of regression slopes. We will use analyses of covariance (ANCOVA) to compare the two groups, which controls for baseline ISI values. We will analyze the treatment effect using the intent-to-treat principle. Descriptive statistics will examine differences in baseline variables between completers and dropouts. If important differences are observed (suggesting the data are missing under a "Not Missing at Random" mechanism) or the loss to follow-up rate exceeds 10%, the primary analysis will be tested through multiple imputation of the ANCOVA model, where potential predictors of dropout will be included in the imputation model, e.g., response to a baseline question asking about the degree of motivation of the participants to stay in the study.

Additional analyses will describe mean changes within groups and test change using paired t-tests. We will also analyze whether we met the clinically relevant target for success of sub-threshold or better scores (≤14) among >80% of the intervention participants. Group differences at post-intervention will be assessed by logistic regression, controlling for baseline ISI score.

Analysis of the Secondary Endpoint(s)

We also plan to calculate differences between study arms for the following ISI subscales: severity of sleep onset, sleep maintenance, early morning awakening, sleep dissatisfaction, interference of sleep with daytime functioning, noticeability of sleep problems by others, and distress caused by sleep difficulties. Each of these items is rated on a 5-point Likert scale. For each outcome, if there is sufficient variation (use of all 5 points), the intervention effect will be tested through ANCOVA, controlling for baseline level. If not, ordinal logistic regression, controlling for baseline will be used. As this is an exploratory analysis and we will not have sufficient power, we will not adjust for Type I error.

Exploratory outcomes:

1) Differences in ISI score by engagement. We will conduct sub-analyses of change in ISI between high engagers (based on frequency of skill utilization) and low engagers (separated by median value).
2) Differences in ISI score by participant satisfaction. Participant satisfaction will be graded on a four-point Likert scale, as well as whether participants would recommend the sleep program to friends or family.
3) We will also explore differences in fatigue and depression (common co-occurring symptoms) as measured on the PROMIS 29, by arm.

Safety Analyses

N/A

Baseline Descriptive Statistics

We will compare intervention and control arms on baseline characteristics using means for continuous variables (age) and frequencies for categorical variables (e.g. stage, education, race/ethnicity, marriage status, employment). Variables such as time since diagnosis or level of physical activity may be analyzed as continuous or categorical depending on the distribution of the data. As recommended by CONSORT guidelines, we will not calculate baseline statistically significant differences by study arm.

Planned Interim Analyses

N/A

Sub-Group Analyses

We will explore data by participant age, race, and recruitment site, if we have appropriate numbers. These analyses will be exploratory in nature.

Tabulation of Individual Participant Data

Individual participant data will not be listed by measure and time point. Rather, it will be presented in the aggregate.

17.1

Data collection will be the responsibility of the MHRI trial staff at the site under Dr. Arem's supervision. All data will be stored within MHRI's secure REDCap system. Dr. Arem will be responsible for ensuring the accuracy, completeness, legibility, and timeliness of the data reported, as well as for storing all informed consent documents and for managing any other identifiable information, including any audio recordings or transcripts.

All study data will be collected in REDCap and non-PHI will be downloaded into a SAS dataset. Study documents will be retained for a minimum of 3 years after the last annual Federal Financial Report (FFR) is submitted. Data will be stored behind the MedStar Health firewall in Box.

Clinical data (including adverse events (AEs), concomitant medications, and expected adverse reactions data) will be entered into REDCap, a 21 CFR Part 11-compliant data

capture system provided by the Medstar Health Research Institute. The data system includes password protection and internal quality checks, such as automatic range checks, to identify data that appear inconsistent, incomplete, or inaccurate. While participants will be recruited from multiple sites, all records will be collected and stored by MHRI and only MHRI research staff who have a need will be able to see identifiable information.

18.0 Provisions to Monitor the Data to Ensure the Safety of Subjects*

N/A. This research involves only Minimal Risk to subjects.

Dr. Arem will have primary responsibility for monitoring the data and safety of this project and complying with the reporting requirements of the funding agency and the IRB of record (MHRI IRB). As part of the annual progress report to the NIH sponsor, the PI will provide an update of project activities to date, summary of the characteristics of participants, expected versus actual enrollment, and any changes to the protocol. The PI will work closely with members of the study team to ensure that a quality assurance plan and the data and safety monitoring plan are thoroughly followed.

Adverse events and Serious Adverse Events

Definition of Adverse Events
This protocol uses the definition of adverse event from 21 CFR 312.32 (a): any untoward medical occurrence associated with the use of an intervention in humans, ***whether or not considered intervention-related***.

Definition of Serious Adverse Events

A serious adverse event (SAE) is any untoward medical occurrence that:

- Results in death.
- Is life-threatening. Any adverse experience that places the subject, in the view of the investigator, at immediate risk of death from the reaction as it occurred (i.e., it does not include a reaction that, had it occurred in a more serious form, might have caused death).
- Requires in-patient hospitalization or prolongation of existing hospitalization.
- Results in persistent or significant disability or incapacity.
- An event that requires intervention to prevent permanent impairment or damage.
- Important medical events that do not result in death, are not life-threatening, or do not require hospitalization may be considered serious adverse events when, based upon appropriate medical judgment, they might jeopardize the subject and might require medical or surgical intervention to prevent one of the outcomes listed above.

Classification of an Adverse Event
Severity of Event
For adverse events (AEs) not included in the protocol defined grading system, the following guidelines will be used to describe severity.

- **Mild** – Events require minimal or no treatment and do not interfere with the participant's daily activities.

- **Moderate –** Events result in a low level of inconvenience or concern with the therapeutic measures. Moderate events may cause some interference with functioning.
- **Severe –** Events interrupt a participant's usual daily activity and may require systemic drug therapy or other treatment. Severe events are usually potentially life-threatening or incapacitating. Of note, the term "severe" does not necessarily equate to "serious".

Relationship to Study INTERVENTION/Experimental Manipulation

All adverse events (AEs) will have their relationship to study procedures, including the intervention, assessed by an appropriately-trained clinician based on temporal relationship and his/her clinical judgment. The degree of certainty about causality will be graded using the categories below.

- **Definitely Related –** There is clear evidence to suggest a causal relationship, and other possible contributing factors can be ruled out. The clinical event, including an abnormal laboratory test result, occurs in a plausible time relationship to study procedures administration and cannot be explained by concurrent disease or other drugs or chemicals. The response to withdrawal of the study procedures should be clinically plausible. The event must be pharmacologically or phenomenologically definitive.
- **Probably Related –** There is evidence to suggest a causal relationship, and the influence of other factors is unlikely. The clinical event, including an abnormal laboratory test result, occurs within a reasonable time after administration of the study procedures, is unlikely to be attributed to concurrent disease or other drugs or chemicals, and follows a clinically reasonable response on withdrawal.
- **Potentially Related –** There is some evidence to suggest a causal relationship (e.g., the event occurred within a reasonable time after administration of study procedures). However, other factors may have contributed to the event (e.g., the participant's clinical condition, other concomitant events). Although an AE may rate only as "possibly related" soon after discovery, it can be flagged as requiring more information and later be upgraded to "probably related" or "definitely related", as appropriate.
- **Unlikely to be related –** A clinical event, including an abnormal laboratory test result, whose temporal relationship to study procedures administration makes a causal relationship improbable (e.g., the event did not occur within a reasonable time after administration of the study procedures) and in which other drugs or chemicals or underlying disease provides plausible explanations (e.g., the participant's clinical condition, other concomitant treatments).
- **Not Related –** The AE is completely independent of study procedures administration, and/or evidence exists that the event is definitely related to another etiology. A clinician will document the alternative, definitive etiology.

Expectedness

A clinician with appropriate expertise in breast cancer and/or CBT-I will be responsible for determining whether an adverse event (AE) is expected or unexpected. An AE will be considered unexpected if the nature, severity, or frequency of the event is not consistent with the risk information previously described for the study procedures.

Time Period and Frequency for Event Assessment and Follow-Up

We do not anticipate adverse event (AE) or serious adverse event (SAE) during the course of this study. However, the principal investigator will be responsible for following adverse event reporting requirements as outlined below. These responsibilities include:

a. Reviewing the accuracy and completeness of all adverse events reported
b. Compliance with IRB policies for reporting adverse events and/or serious adverse events
c. Closely monitoring research participants at each point of contact for any new Adverse Events (AEs) or Serious Adverse Events (SAEs).

All AEs, not otherwise precluded per the protocol, will be captured on the appropriate case report form (CRF). Information to be collected includes event description, time of onset, assessment of severity, relationship to study procedures, and time of resolution/stabilization of the event. All AEs occurring while on study will be documented appropriately regardless of relationship. All AEs will be followed to adequate resolution.

Any medical or psychiatric condition that is present at the time that the participant is screened will be considered as baseline and not reported as an AE. However, if the study participant's condition deteriorates at any time during the study, it will be recorded as an AE. Changes in the severity of an AE will be documented to allow an assessment of the duration of the event at each level of severity to be performed.

Adverse Event Reporting

Any mild to moderate event on the above scale will be reported by the MHRI PI to the IRB within 5 business days.

Serious Adverse Event Reporting

1. The study is of low to moderate risk. No serious adverse events are expected.

2. We will attempt to report serious unanticipated events within 24 hours to the GU/MHRI IRB, but no longer than 3 business days. Adverse events will be considered serious if graded 3 or higher on the scale outlined above.

3. An adverse event report including a description of the event, when the event occurred, and when and how the event was reported, will be generated for each event. Any documentation related to the event or its attribution will be included in the report.

4. The study PI point of contact with the IRB (Dr. Arem) will conduct a review of all adverse events upon completion of every study subject. She will evaluate the frequency and severity of the adverse event(s) and, in conjunction with the IRB at GU/MHRI, will determine if modifications to the protocol or consent forms are required.

The IRB will evaluate whether the study should continue unchanged, require modification, continue, or close to enrollment. If increased frequency of serious adverse events is detected and reported, an ad hoc safety review will be convened to determine whether and how to modify, continue, or terminate enrollment.

19.0 Provisions to Protect the Privacy Interests of Subjects

All participant data recorded by the Alexa Echo Dot device or sent to the Alexa Echo during the course of the testing is encrypted for patient privacy. All participant data collected by the Alexa Echo system is held on Amazon's servers in a de-identified form. All participant data to and from the smartphone app will likewise be encrypted and will not reside on patient smartphones, but will only be held on protected servers in a de-identified format. Media Rez will make this de-identified data available to MHRI for data analysis. GW MHRI holds the keys to participant data identification, but at no time will any identified or identifiable participant data be sent to or stored at Media Rez. All participants will use Alexa accounts created by Media Rez to prevent Amazon from gaining access to patient names or privacy data.

All staff will be trained on MedStar Health standards of confidentiality prior to being given access to any data using the CITI training certification. Staff training is comprehensive and will entail not only confidentiality but also respect for persons, safety, the intervention, testing, and all related components of the study. The PI will oversee and ensure ongoing respect for participants and their data throughout the study.

Study staff will be trained on confidentiality standards and proper interviewing technique. This will include the following points:

- Always protect participant data and confidentiality of persons.
- Participant names should not be included in any data collection instruments or systems. The only numbers used to label and identify data from the participant are the Survey ID or the Recruiter ID.
- Questionnaires and any digital recordings are to be linked using the Survey ID number and the interview date. No personal identifiers should be written or affixed to the survey results.
- Protect the electronic security of all databases.
- Computers that can access electronic data should be physically secured and should be protected by coded passwords. Networks must be demonstrated to be secure by the IT department prior to use.
- Only authorized persons are to have access to electronic databases.
- Project staff must shred documents containing sensitive information before disposing of them.

Other concerns and resolutions to specific to using the Amazon Alexa device:
Delete Voice Recordings: One risk is that every time the participant says the wake word "Alexa," the participant's voice is recorded and sent to Amazon's servers, where Amazon could potentially misuse the voice recordings because these recordings are potentially identifiable. While these voice recordings are not available to anyone outside of Amazon, there is a small chance that they will be somehow misused by Amazon. To mitigate this small but real risk, Media Rez will delete these voice recordings on a daily basis.

Further, Media Rez will delete them via Amazon's content management tools without hearing them. Because Alexa accounts are controlled by Media Rez, participants will not have to sign up with Amazon or provide Amazon with any identifiable data.

Disable Calling: Another risk posed by using an Alexa Echo Dot is the chance of being overheard by other Echo owners by accidently connecting to them via calling and messaging. To prevent this (which has happened to at least one Echo owner who inadvertently sent voice recordings to people in their contracts list), Media Rez will disable these calling and messaging features for the duration of the testing.

Disable Voice Purchasing: Likewise, voice-based purchasing will be turned off (and will not be linked to the participant or their credit cards or their Amazon account).
Mute Mode: We will also inform participants about how they can mute the Echo Dot by pressing the mute button.

We believe that these risks are minimal and are outweighed by the potential benefit of improved sleep.

20.0 Compensation for Research-Related Injury

20.1 We believe that this study involves only Minimal Risk to subjects. We will not provide any compensation in the event of research-related injury.

21.0 Economic Burden to Subjects

21.1 Subjects will be responsible for all regular care, but not for any expenses related to the study. We will mail study devices to participants and will provide return shipping labels.

22.0 Consent Process

Informed Consent Process

All informed consent will be conducted in compliance with NIH Human Research Protections Program policies and procedures. A study team member trained in human subjects research will conduct informed consent as described below:

Consent/assent and Other Informational Documents Provided to participants

We will have 2 separate consent forms: a) for screening and b) for the RCT.

Consent forms describing in detail the study intervention, study procedures, and risks will be given to the participant and written documentation of informed consent will be completed prior to starting the study intervention.

Consent Procedures and Documentation

Informed consent will be conducted by phone. Anyone called will first be read the consent for screening form, which requires only a verbal consent so as to minimize the potential to link PHI. Individuals will be asked a series of questions by a MHRI research assistant or clinical research coordinator to determine eligibility (including the Insomnia Severity Index), and will be notified of whether they are eligible within 3 business days.

If deemed eligible, participants will receive a copy of the full consent form by email. The CRC will then set up a phone call where study staff will review the full consent form and ask individuals if they have any questions or concerns. Once informed consent has been completed, participants will receive a subject ID, which will be linked to a personalized survey administered via REDCap. Participants will also be able to view a written version of the informed consent on a landing page in REDCap, which they must agree to before continuing to complete baseline forms. The landing page will include a single page summary of the research, with an option to download the full informed consent again (although they will have previously received the full form by email). Both the text that will be on the landing page and the full informed consent are included in this application.

Confidentiality and Privacy

Participant confidentiality and privacy is strictly held in trust by the participating investigators, their staff, the safety and oversight monitor(s), and the sponsor(s) and funding agency. This confidentiality is extended to the data being collected as part of this study. Data that could be used to identify a specific study participant will be held in strict confidence within the research team. No personally-identifiable information from the study will be released to any unauthorized third party without prior written approval of the sponsor/funding agency.

All research activities will be conducted in as private a setting as possible.

The study monitor, other authorized representatives of the sponsor or funding agency, representatives of the Institutional Review Board (IRB), regulatory agencies or representatives from companies or organizations supplying the product, may inspect all documents and records required to be maintained by the investigator, including but not limited to, medical records (office, clinic, or hospital) and pharmacy records for the participants in this study. The clinical study site will permit access to such records.

In this study, Media Rez provides Amazon Alexa Echo Dot devices to individuals who have met eligibility criteria and agreed to participate. All participant data recorded by the Alexa Echo Dot device or sent to the Alexa Echo during the course of the testing is encrypted for patient privacy. All participant data collected by the Alexa Echo system is held on Amazon's servers in a de-identified form. All participant data to and from the smartphone app will likewise be encrypted and will not reside on patient smartphones, but will only be held on protected servers in a de-identified format. Furthermore, Media Rez provides both the phone and the hotspot so that the individual does not have to link the device to her personal wifi. Media Rez will make this de-identified data available to MHRI for data analysis. MHRI maintains participant data identification. At no time will any identified or identifiable participant data be sent to or stored at Media Rez.

The study participant's contact information will be securely stored at MHRI for internal use during the study. At the end of the study, all records will continue to be kept in a

secure location for as long a period as dictated by the reviewing IRB, Institutional policies, or sponsor/funding agency requirements.

Study participant research data, which is for purposes of statistical analysis and scientific reporting, will be stored at MHRI. This will not include the participant's contact or identifying information. Rather, individual participants and their research data will be identified by a unique study identification number. The study data entry and study management systems used by clinical sites and by MHRI research staff will be secured and password protected. At the end of the study, all study databases will be de-identified and archived at MHRI.

Measures Taken to Ensure Confidentiality of Data Shared per the NIH Data Sharing Policies
It is NIH policy that the results and accomplishments of the activities that it funds should be made available to the public (see https://grants.nih.gov/policy/sharing.htm). The PI will ensure all mechanisms used to share data will include proper plans and safeguards for the protection of privacy, confidentiality, and security for data dissemination and reuse (e.g., all data will be thoroughly de-identified and will not be traceable to a specific study participant). Plans for archiving and long-term preservation of the data will be implemented, as appropriate.

- **Future Use of Data**

Data collected for this study will be analyzed and stored at MHRI for five years after trial completion. We will make de-identified relevant data and associated documentation available to users who request access to the data one year after study completion only under a data sharing agreement that provides for: (1) a commitment to using the data only for research purposes and not to seek to identify any individual participant; (2) a commitment to securing the data using appropriate computer technology; and (3) a commitment to destroying or returning the data after analyses are completed.

23.0 Process to Document Consent in Writing

Any in-person consenting will include written consent as follows: A study team member who has completed human subjects training will read through informed consent with patient participants and will answer any questions about the study. The patient will then be asked if he or she would like more time to consider the study or if he or she would prefer to go ahead and complete the informed consent. If the informed consent is signed in person, the individual obtaining consent will sign as well. In the case that the individual is consented remotely (due to pandemic conditions, his/her preferences, the location of the participant, or scheduling), we will first email (or mail if preferred) a copy of the informed consent to the participant. We will then schedule a time to read through the consent by phone or Microsoft Teams to give them a chance to ask any questions about the study. We will verbally confirm whether they want to proceed. If they agree, we will send them an individualized REDCap link with baseline forms. The REDCap landing page will have the summary of the informed consent (the brief version), with a link to the detailed ICF document, and will ask the participant to write their name to proceed. REDCap has an e-consent module where participants type their name and certify

consent before being allowed to proceed with data collection. REDCap's e-consent process has two steps: 1) Before a participant completes the survey, an extra certification page is added to the end of the survey that displays an in-line PDF copy of the document in which they will be asked to confirm that personal information (name) in the document is correct. This survey will not be considered complete until they fulfill the certification step. 2. Upon completion of the survey, a static copy of their responses in the form of a consent-specific PDF will be stored in the project's File Repository.

24.0 Setting

Participants will be recruited from MedStar Washington Hospital Center and Georgetown's Lombardi Comprehensive Cancer Center from the breast oncology clinics. The clinical research coordinator (CRC) will use MedConnect and will work with the breast oncology and survivorship care clinics as needed to identify potentially eligible subjects and to flag these participants for the oncologist. With oncologist approval, the CRC will reach out to patients by phone to describe the trial and if the patient is interested, to conduct informed consent and baseline forms. All forms can be completed online by the participant, or conducted by phone with the CRC, or if preferred by participants, can be mailed in hard copy to the individual.

The intervention delivery and data collection will be conducted remotely. Primary settings for recruitment include the Medstar Washington Hospital Center, Georgetown's Lombardi Comprehensive Cancer Center, and the Dana-Farber Cancer Institute (DFCI)/Harvard Cancer Center. Each will abide by site-specific preferences around recruitment, and will work with physicians on site to lower the burden on physicians. If recruitment is slow, we will submit a modification to expand to other MedStar facilities including MedStar Franklin Square Hospital, and/or MedStar Union Memorial Hospital.

25.0 Resources Available

The research team (Dr. Arem, Claire Starling, and collaborators) will meet weekly to discuss ongoing research progress. Internally at MHRI, we will conduct additional weekly meetings to discuss participant recruitment and any updates to research protocol or duties and functions. Dr. Arem is dedicating 20% effort to the project, and Claire Starling, research coordinator will dedicate 50-100% effort as needed. We will also include breast medical oncologist Dr. Christopher Gallagher at 2% effort and an oncology nurse at 5% effort to support recruitment efforts. Collaborators at Media Rez (prime) and Harvard/DFCI also have dedicated effort towards developing this project.

Data from the WHC Medical Records indicate that 275 women per year on average are treated at WHC for breast cancer; numbers are similar for GU. We anticipate that ~30% of these women will have insomnia after treatment completion. Given that we are including women who are survivors, women who return for adjuvant therapy for 5-10 years post-curative treatment will be eligible for screening, increasing our sample size.

We do not anticipate medical or psychological resources needed for patients, but will refer them to their primary care physician for these issues- no services will be provided through our research study.

26.0 Multi-Site Research*

While we are recruiting from multiple sites, all data will be collected and stored at MHRI. Thus, no data will be held by other sites.
