# Supplementary material for: Voice-Activated Cognitive Behavioral Therapy for Insomnia: A Randomized Clinical Trial
Source: JAMA Netw Open. 2024 Sep 24;7(9):e2435011. doi: 10.1001/jamanetworkopen.2024.35011 (PMC11423177; doi:10.1001/jamanetworkopen.2024.35011)
Supplement: Supplement 2. — Data Sharing Statement [file jamanetwopen-e2435011-s002.pdf]

Data Sharing Statement

Starling. Voice-Activated Cognitive Behavioral Therapy for Insomnia. *JAMA Netw Open*. Published September 24, 2024. doi:10.1001/jamanetworkopen.2024.35011

Data
Data available: Yes
Data types: Deidentified participant data, Data dictionary
How to access data: Data sharing is available upon reasonable request from study authors at Hannah.Arem@medstar.net.
When available: With publication

Supporting Documents
Document types: Informed consent form
How to access documents: Informed consent will be provided upon reasonable request to corresponding author Hannah.Arem@medstar.net
When available: With publication

Additional Information
Who can access the data: Researchers whose proposed use of the data is reasonable.
Types of analyses: For related scientific work.
Mechanisms of data availability: Data will be available with a signed data access agreement.
